# Associations between care home residents’ characteristics and acute hospital admissions – a retrospective, register-based cross-sectional study

**DOI:** 10.1186/s12877-023-03895-1

**Published:** 2023-04-18

**Authors:** Gitte Schultz Kristensen, Anette Hvenegaard Kjeldgaard, Jens Søndergaard, Karen Andersen-Ranberg, Andreas Kristian Pedersen, Christian Backer Mogensen

**Affiliations:** 1 Emergency Department, Aabenraa Hospital, Department of Regional Health Research, Faculty of Health Science, University Hospital of Southern Jutland, University of Southern Denmark, Odense, Denmark; 2 Department of Medicine, Aabenraa Hospital, University Hospital of Southern Jutland, Jutland, Denmark; 3 Research Unit of General Practice, Department of Public Health, University of Southern Denmark, Odense, Denmark; 4 Geriatric Research Unit, Department of Clinical Research, Department of Public Health, Department of Regional Health Research, Faculty of Health Science, Clinical research Department, Aabenraa Hospital, University of Southern Denmark University Hospital of Southern Denmark, Odense, Denmark; 5 Department of Regional Health Research, Faculty of Health Science, Emergency Department, Aabenraa Hospital, The University of Southern Denmark, University Hospital of Southern Denmark, Odense, Denmark

**Keywords:** Care home, Nursing home, Acute admission, Hospitalisation, Prevention, Morbidity, Survival, Predictors, Descriptive study, Register-based.

## Abstract

**Background:**

Care home residents are frail, multi-morbid, and have an increased risk of experiencing acute hospitalisations and adverse events. This study contributes to the discussion on preventing acute admissions from care homes. We aim to describe the residents’ health characteristics, survival after care home admission, contacts with the secondary health care system, patterns of admissions, and factors associated with acute hospital admissions.

**Method:**

Data on all care home residents aged 65 + years living in Southern Jutland in 2018–2019 (n = 2601) was enriched with data from highly valid Danish national health registries to obtain information on characteristics and hospitalisations. Characteristics of care home residents were assessed by sex and age group. Factors associated with acute admissions were analysed using Cox Regression.

**Results:**

Most care home residents were women (65.6%). Male residents were younger at the time of care home admission (mean 80.6 vs. 83.7 years), had a higher prevalence of morbidities, and shorter survival after care home admission. The 1-year survival was 60.8% and 72.3% for males and females, respectively. Median survival was 17.9 months and 25.9 months for males and females, respectively. The mean rate of acute hospitalisations was 0.56 per resident-year. One in four (24.4%) care home residents were discharged from the hospital within 24 h. The same proportion was readmitted within 30 days of discharge (24.6%). Admission-related mortality was 10.9% in-hospital and 13.0% 30 days post-discharge. Male sex was associated with acute hospital admissions, as was a medical history of various cardiovascular diseases, cancer, chronic obstructive pulmonary disease, and osteoporosis. In contrast, a medical history of dementia was associated with fewer acute admissions.

**Conclusion:**

This study highlights some of the major characteristics of care home residents and their acute hospitalisations and contributes to the ongoing discussion on improving or preventing acute admissions from care homes.

**Trial registration:**

Not relevant.

**Supplementary Information:**

The online version contains supplementary material available at 10.1186/s12877-023-03895-1.

## Background

As in many countries, Danish care home residents (CHRs) represent the frailest, most vulnerable citizens in society [1, 2]. Citizens admitted to care home often suffer from several concomitant diseases, and studies show that new CHRs today have greater multi-morbidity and more complex care needs than 15–20 years ago [2–5]. Furthermore, advanced age and multi-morbidity are associated with an increased risk of experiencing acute, unplanned hospital admissions, and residents of care home facilities have a higher rate of hospital admissions than their community-dwelling peers [2, 6, 7].

In general, acute hospital admissions of CHRs are often prolonged and more costly than admissions of older people from the wider community [8, 9], due to a higher risk of adverse events such as hospital-acquired infections, delirium, falls, fractures, and loss of functional capability and self-dependence [10, 11]. These adverse events threaten to leave the resident even frailer after hospital discharge. If some of the unplanned admissions from care homes could be prevented, it might serve a dual benefit by improving residents’ conditions and lowering healthcare costs [12].

Studies show that up to 67% of acute hospital admissions from care homes could potentially have been managed in the primary sector and thereby prevented [10, 12, 13]. However, these assessments of the frequency of potentially preventable admissions are theoretical and based on retrospective reviews of medical records. Clinical studies on preventing hospitalisations from care homes often show low quality, and interventions differ considerably [14, 15]. Furthermore, international studies on CHRs and acute admissions from care homes are not necessarily comparable with Danish or Scandinavian conditions due to substantial variations in the organisation of care facilities and the services of personal care they provide. Additionally, as many countries lack a national register on CHRs, studies on acute admissions from care homes often only include residents admitted to hospital and cannot identify and compare to residents with no hospital admissions. Denmark’s complete register of all CHRs provides a unique opportunity to characterize the residents and their use of health care services [16, 17]. If healthcare professionals can identify residents with an increased risk of acute admission, it will be more straightforward to target a preventive effort. We need to know more about the CHRs, their health characteristics, admission patterns, and factors associated with acute admissions.

This retrospective register-based cross-sectional study aims to provide evidence-based information to the ongoing discussion on preventing acute hospital admissions of CHRs. Specifically, our objectives are to describe the care home residents’ characteristics, survival after care home admission, contacts with the secondary health care system, patterns for admissions, and to identify factors associated with acute hospital admissions.

## Methods

### Study population and data sources

We studied the extent of contact with the secondary healthcare system among CHRs living in Southern Jutland, Denmark, from 1st January 2018 to 31st December 2019. We included all citizens aged 65 years or older who lived permanently or moved into a care home facility during the two-year study period. The cohort was enriched with individual-level data on hospital admissions, emergency room visits, and the use of prescription medicines. Data were assembled from the data sources seen in Table 1.

**Table 1:** Description of data sources

Data source	Description
Care Home Data (CHData)	We identified the study population through CHData, which contains highly valid information on all Danish citizens affiliated with a care home address from 2014 to the present [16, 17]. All care home residents in Southern Jutland were identified with a CPR number (civil registration number), a care home name, and a date of care home admittance. The CPR number is a unique 10-digit identifier assigned to all Danish citizens, which serves as a link to other national registers [18].
The Danish Civil Registration System (CRS)	Data on birthdate, date of death or migration (if relevant), and gender were obtained from the CRS, which contains general information on the entire Danish population since 1968 [18].
The Danish National Patient Register (DNPR)	All data on in- and outpatient hospital diagnoses, admissions, and emergency room visits were obtained from the DNPR [19]. This register contains information on all non-psychiatric hospital admissions since 1977 and all inpatient and outpatient contacts to the secondary health care system since 1995, psychiatric and somatic [19]. For every contact, one primary and optional secondary diagnosis are recorded according to the International Classification of Diseases (ICD-10).
The Danish National Prescription Registry (NPR)	From the NPR, we obtained information on filled prescriptions. This register contains individual-level data on all dispensing of prescription medicine since 1995 [20], and drugs are categorised according to the Anatomical Therapeutic Chemical index (ATC-codes) [21].

### Setting

All 98 Danish municipalities are responsible for local health and social services, including disease prevention, home care services, rehabilitation, and care home facilities [22, 23]. Citizens become eligible for a care home residency when extensive care is required due to permanent and substantial impairment of physical or mental function. The municipalities appoint residency to the citizens in greatest need, regardless of financial means. The municipalities manage access to residency in both private and public care homes, and all facilities are subject to the Danish Law on Social Services [24]. The care home facilities are staffed 24 h a day with nurse assistants and/or other healthcare professionals with 1.6–3.3 years of education, supplemented with unskilled workers, and nurses during the daytime [7]. In addition, community nurses are on call during evenings, nights, and weekends.

The geographical part of Denmark, known as Southern Jutland, comprises four municipalities with approximately 225,000 inhabitants in rural and urban areas, hereof 40,108 aged 65–79, 11,043 aged 80–89, and 2,048 aged 90 + years in 2019, and demographics are similar to the Danish population [25]. In 2019, residents in care home facilities accounted for 0.67% of citizens living in Southern Jutland, comparable to 0.69% of all Danish citizens [26]. In 2019 the four municipalities managed 38 care home facilities with around 1600 long-term beds in total [27].

The Danish healthcare system is tax-funded and offers all citizens free and equal access to healthcare. Primary care physicians (PCPs) handle most medical problems and manage all hospital referrals as gatekeepers to the secondary healthcare system (except in medical emergencies) [22, 23]. The Emergency Department (ED) covers the Emergency Room (ER), where orthopaedic injuries and medical emergencies are managed, and the acute admissions ward, managing other acute hospital referrals (e.g., acute medical, neurological, or surgical patients referred from PCPs). When admitted to hospital, most patients are received in the ED. Patients with pre-hospital identified acute cardiovascular disease or ongoing oncological treatment are exceptions. From the ED, patients are either discharged home, admitted within the ED (patients expected to have a short admission ≤ 48 h), or transferred to an in-hospital ward (patients with expected > 48 h of admission).

In this study, we regarded all ER visits with no further need for hospitalisation as acute outpatient contacts. This included all visits due to minor injuries such as wounds, contusions, distortions, and fractures managed within the ER. Acute admissions were defined as all other unplanned hospital contacts regardless of duration, such as cases of hip fractures, pneumonia, urinary tract infection, or dehydration, leading to a short admission within the ED or a more prolonged admission in a hospital ward. The inclusion and exclusion criteria in the definition of acute hospital admissions are depicted later in this report.

### Data variables and analysis

We described residents in terms of sex, age at the time of care home admission, and selected morbidities. We divided residents into three age groups based on their age on 1st January 2018. Selected morbidities were assessed by collecting all primary and secondary ICD-10 diagnoses assigned to all in- and outpatient hospital contacts during the past ten years from baseline (either 1st January 2018 or date of care home admission if later than this). The ICD-10 diagnoses were combined with data on the use of prescription medicines (ATC-codes) during the past year from baseline. Residents were coded as having the morbidity of interest if they presented with a relevant ICD-10 code in the hospital records and/or were users of prescription medicines indicated for that specific disease. To avoid overestimating the disease prevalence, ATC-codes were not included in the assessment of morbidities if the particular drug had several indications. The use of information from ICD-10 codes and ATC-codes in forming the selected morbidities is described in detail in Additional file 1. The prevalence of each selected morbidity is presented as totals and proportions, and the sum of selected morbidities is presented as numbers and medians with interquantile range (IQR). All characteristics were assessed in total as well as stratified by each age and sex category. For residents admitted to care homes during the study period, mortality after care home admission was described using a Kaplan-Meier curves with a follow-up period of a maximum of four years.

Next, the incidences of hospitalisations and emergency room visits in 2018–2019 were calculated based on the resident-time at risk. We estimated the resident-time at risk by calculating the mean duration of care home stays in 2018–2019. The duration of stay corresponds to the resident-time at risk of being in contact with the health care system as a CHR. Finally, we calculated the incidences of hospital contacts by dividing the number of contacts by the number of resident-years in total and by each age and sex category.

Furthermore, we described all acute hospital admissions of CHRs in 2018–2019 in terms of day of admission, time of the day, destination (discharged from ED to care home or transferred to an in-hospital ward), inpatient days, length of admission (≤ 48 h or > 48 h), diagnoses at discharge, acute readmissions within 30 days, in-hospital mortality, and 30-days mortality post-discharge. The diagnoses at discharge were assessed using only the primary discharge diagnoses, and ICD-10 codes were categorized into subgroups within the ICD-10 chapters, as shown in Additional file 2. Inpatient days are measured as median with IQR, while the remaining results are presented as total and proportions.

Finally, we divided the cohort into two groups; residents who experienced at least one acute hospital admission during the study period and residents with no acute admissions. The relation between specific characteristics of the residents and acute hospital admissions was analysed using Cox Regression, adjusting for the left-truncation set on 1st January 2018 and adjusting for competing risks by viewing it as censoring. Admission was the event of interest, and death due to any cause was the competing event preventing the resident from experiencing the event of interest. Results are presented as unadjusted and adjusted cause-specific hazard ratios (HR) with 95% confidence intervals (CI). In the multivariate analysis, we adjusted for possible confounders concerning the given exposure based on clinical knowledge, as shown in Additional file 3.

No data was missing. The Danish Health Data Authority provided all data in the present study. Using Stata version 17.0, data was processed on the Danish Health and Medicines Authority’s Research Machine (Forskermaskinen). The processing of personal data in the present study is notified to and approved by the Region of Southern Denmark and listed in the internal record (19/432,119) cf. Art 30 of The General Data Protection Regulation. According to Danish law, studies based solely on register data do not require approval from an ethics committee or informed content from the study participants [28].

## Results

A total of 2601 citizens aged 65 + years resided permanently in a care home facility in Southern Jutland in 2018–2019, of which 65.6% were women, and the mean age at care home admission was 82.7 years (83.7 and 80.6 years for females and males, respectively). Male residents tended to have a higher prevalence of morbidities than females. Most of the selected morbidities were more frequently registered in the younger age groups, e.g., diabetes, dementia, Parkinson’s disease, stroke, alcohol abuse, and chronic obstructive pulmonary disease (COPD) or asthma. In contrast, other morbidities showed an inverse relationship with age group, e.g., atrial fibrillation and ischemic heart disease; see Table 2.

**Table 2:** Characteristics of all care home residents aged 65 + years living in Southern Jutland in 2018–2019. Overall, as well as stratified by sex and age group

		Total	Female	Male	Age 65–79	Age 80–89	Age 90+
Care home residents		2601	1705 (65.6%)	896 (34.4%)	769 (29.6%)	1194 (45.9%)	638 (24.5%)
Age at care home admission		82.7	83.7	80.6	-	-	-
Resident-years in care home during the study period		3017.2	2044.4	972.8	977.0	1322.2	717.9
Selected morbidities							
	Cancer	506 (19.5%)	303 (17.8%)	203 (22.7%)	154 (20.0%)	239 (20.0%)	113 (17.7%)
	Diabetes	476 (18.3%)	278 (16.3%)	198 (22.1%)	163 (21.2%)	235 (19.7%)	78 (12.2%)
	Dementia	1300 (50.0%)	856 (50.2%)	444 (49.6%)	409 (53.2%)	644 (53.9%)	247 (38.7%)
	Parkinson’s disease	136 (5.2%)	73 (4.3%)	63 (7.0%)	77 (10.0%)	53 (4.4%)	6 (0.9%)
	Alcohol abuse	166 (6.4%)	64 (3.8%)	102 (11.4%)	122 (15.9%)	42 (3.5%)	2 (0.3%)
	Schizophrenia, schizotypal and delusional disorders	81 (3.1%)	61 (3.6%)	20 (2.2%)	47 (6.1%)	25 (2.1%)	9 (1.4%)
	Mood disorders	411 (15.8%)	295 (17.3%)	116 (13.0%)	160 (20.8%)	180 (15.1%)	71 (11.1%)
	Anxiety	147 (5.7%)	95 (5.6%)	52 (5.8%)	78 (10.1%)	57 (4.8%)	12 (1.9%)
	Hypertension	1394 (53.6%)	908 (53.3%)	486 (54.2%)	360 (46.8%)	682 (57.1%)	352 (55.2%)
	Ischemic heart disease	437 (16.8%)	256 (15.0%)	181 (20.2%)	92 (12.0%)	216 (18.1%)	129 (20.2%)
	Heart failure	274 (10.5%)	157 (9.2%)	117 (13.1%)	52 (6.8%)	150 (12.6%)	72 (11.3%)
	Atrial fibrillation	551 (21.2%)	327 (19.2%)	224 (25.0%)	113 (14.7%)	282 (23.6%)	156 (24.5%)
	Stroke	640 (24.6%)	337 (19.8%)	303 (33.8%)	229 (29.8%)	305 (25.5%)	106 (16.6%)
	COPD*/asthma	444 (17.1%)	279 (16.4%)	165 (18.4%)	148 (19.3%)	213 (17.8%)	83 (13.0%)
	Osteoporosis	612 (23.5%)	511 (30.0%)	101 (11.3%)	146 (19.0%)	322 (27.0%)	144 (22.6%)
Number of selected morbidities							
	Median (IQR)	3 (2–4)	3 (2–4)	3 (2–4)	3 (2–4)	3 (2–4)	2 (1–3)
	0	145 (5.6%)	107 (6.3%)	38 (4.3%)	31 (4.0%)	45 (3.8%)	69 (10.8%)
	1–2	992 (38.1%)	663 (38.9%)	329 (36.7%)	286 (37.2%)	439 (36.8%)	267 (41.8%)
	3–4	1025 (39.4%)	679 (39.8%)	346 (38.6%)	315 (41.0%)	485 (40.6%)	225 (35.3%)
	5+	439 (16.9%)	256 (15.0%)	183 (20.4%)	137 (17.8%)	225 (18.8%)	77 (12.1%)

* Chronic obstructive pulmonary disease

**Fig. 1:**
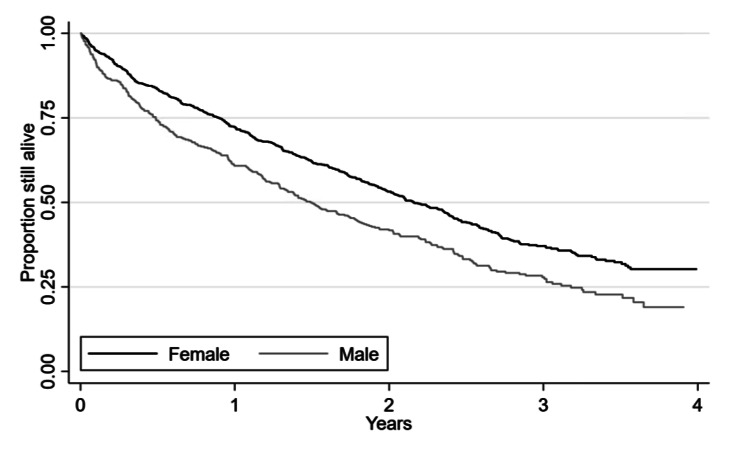
Survival after care home admission for the 1079 individuals admitted to care home during 2018–2019, stratified by sex

During 2018–2019 a total of 1079 individuals were admitted to care homes (388 males and 691 females). Of those, 718 died during the maximum follow-up of four years. The 1-year survival after care home admission was 68.2%, with males having a poorer 1-year survival than females (60.8% and 72.3%, respectively), see Fig. 1. The overall median survival after care home admission was 23.2 months, with 17.9 months for males and 25.9 months for females.

**Fig. 2:**
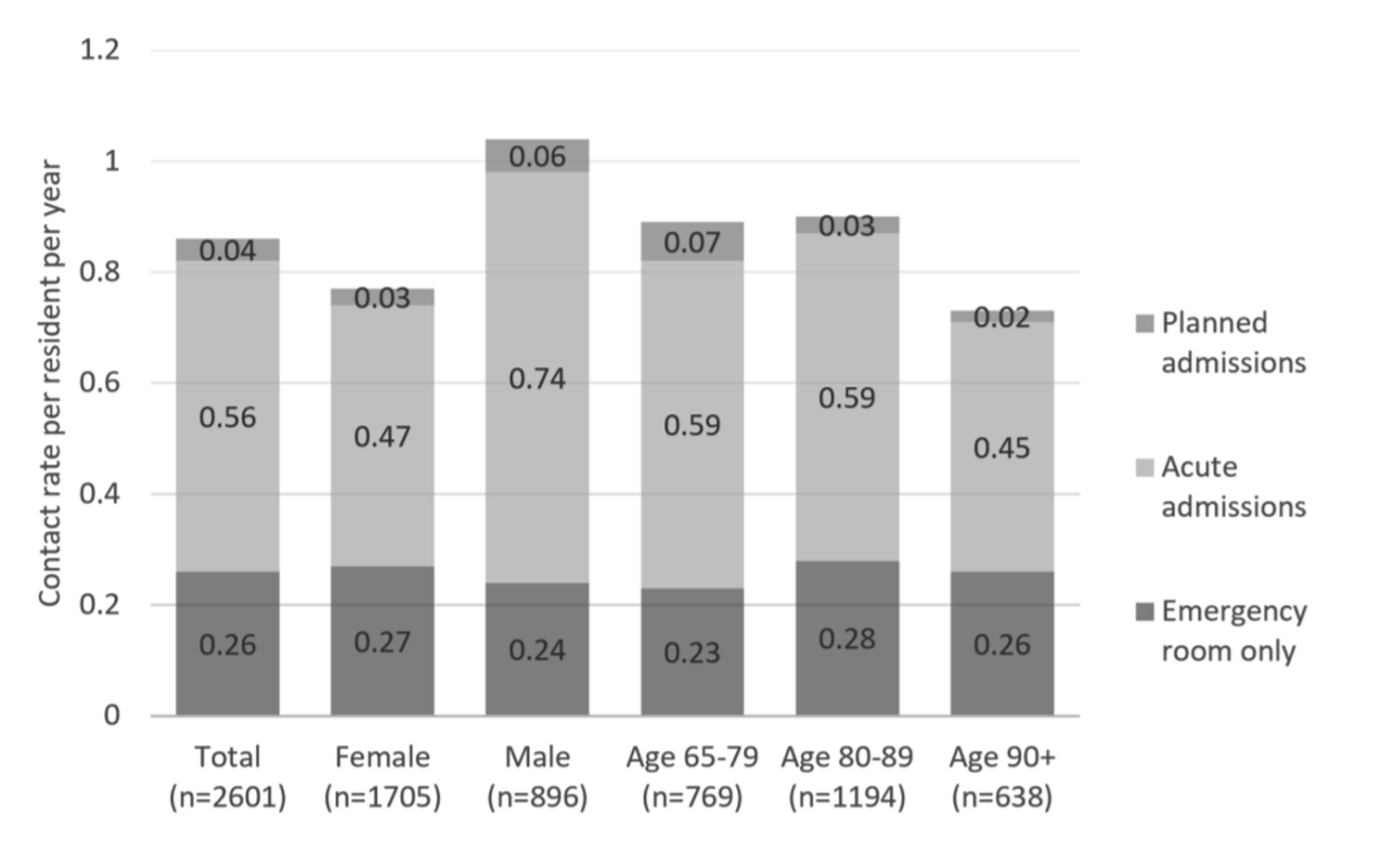
Annual rates of contacts to the secondary health care sector among care home residents aged 65 + years living in Southern Jutland in 2018–2019

The mean rate of acute hospital admissions for all CHRs in the cohort was 0.56 per resident-year, while the emergency room visit rate was 0.26 per resident-year, accounting for all contacts managed in the emergency room not needing further hospitalisation. The rate of planned hospital admissions was only 0.04 per resident-year. The annual rates of acute and planned hospital admissions varied among males and females, with male residents experiencing more hospital admissions per year than females. The admissions rate descended in the highest age group (Fig. 2).

**Fig. 3:**
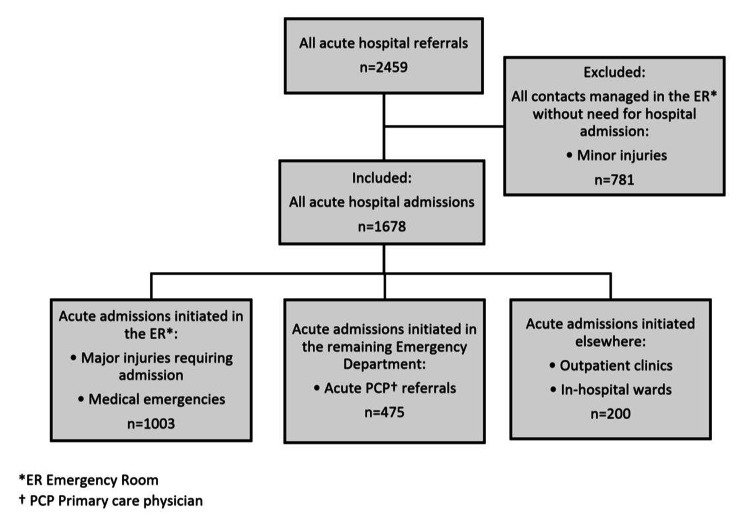
Showing in- and exclusion criteria in the assessment of all acute hospital admissions

During the study period, we observed 2459 acute hospital referrals from care homes in Southern Jutland. Of these, 781 referrals were categorized as acute outpatient contacts, as they were managed solely in the ER with no need for further hospitalisation. The remaining 1678 acute hospital admissions were initiated in the ER, in other wards within the ED (e.g., medical or neurological ED) or elsewhere, e.g., planned outpatient visits converted to an acute admission or patient admitted directly to an in-hospital ward (see Fig. 3). The 1678 acute admissions represent 1032 unique citizens residing permanently in a care home facility in Southern Jutland in 2018–2019. Most admissions occurred during weekdays and dayshifts. About three-fifths (58.5%) of acute admissions lasted more than 48 h, while almost one in four cases (24.4%) CHRs were discharged from the hospital within 24 h. A third (34.4%) of acute admissions were managed solely in the ED without requiring referral to an in-hospital ward. Median inpatient days for all acute admissions were 3 (IQR 1–6). The median inpatient days for the 1101 admissions involving a stay in an in-hospital ward was 5 (IQR 3–8).

The top three primary discharge diagnoses of the 1678 acute admissions were pneumonia, urinary tract infection, and fracture of lower limbs, accounting for 26.9% of all discharge diagnoses. A complete description of all primary discharge diagnoses from acute admissions is found in Additional file 2. Readmission within 30-days occurred in 24.6% (48.2% within seven days; 70.9% within 14 days post-discharge). The primary discharge diagnoses of the acute readmissions were similar to those found at the index admissions, as shown in Additional file 4. A detailed description of all acute hospital admissions, incl. readmissions, is presented in Table 3.

**Table 3:** All acute hospital admissions of care home residents aged 65 + years living in Southern Jutland in 2018–2019

Acute admissions in 2018–2019: n = 1678			n (%)
Duration			
	Short ≤ 48 h		697 (41.5%)
	Long > 48 h		981 (58.5%)
Day of admission			
	Weekday (Monday-Friday)		1289 (76.8%)
	Weekend (Saturday-Sunday)		389 (23.2%)
Time of admission			
	Day-shift (08.00-15.59)		966 (57.6%)
	Evening (16.00-23.59)		546 (32.5%)
	Night (00.00-07.59)		166 (9.9%)
Destination			
	Emergency Department only		577 (34.4%)
	Transferred to ward (n = 901) or admitted directly to an in-hospital ward (n = 200):		1101 (65.6%)
		Internal medicine	582 (34.7%)
		Orthopaedic surgery	169 (10.1%)
		Abdominal surgery	95 (5.6%)
		Neurology	91 (5.4%)
		Cardiology	73 (4.4%)
		Psychiatric	55 (3.3%)
		Other	36 (2.1%)
Most frequent primary discharge diagnoses			
	Pneumonia (J13-J18)		221 (13.2%)
	Fractures of lower limbs (S72, S82, S92)		119 (7.1%)
	Urinary tract infections (N30, N390)		111 (6.6%)
	Other bacterial diseases (A3-A4)		100 (6.0%)
	Volume depletion or electrolyte disorders (E86-E87)		86 (5.1%)
	Respiratory failure, not elsewhere classified (J96)		54 (3.2%)
	Chronic lower respiratory diseases (J40-J47)		51 (3.0%)
	Symptoms, signs, and abnormal clinical and laboratory findings (R00-R99)		162 (9.7%)
	Medical observation and evaluation for suspected diseases and conditions (Z03)		81 (4.8%)
	Other		693 (41.3%)
Readmission within 30 days			413 (24.6%)
In-hospital mortality			183 (10.9%)
Mortality ≤ 30 days post-discharge			218 (13.0%)

When investigating CHRs with and without acute hospital admission, we found that the strongest predictors of acute admission were male gender (HR 1.49) and medical history of heart failure (HR 1.38), diabetes (HR 1.32), COPD/asthma (HR 1.30), hypertension (HR 1.28), atrial fibrillation (HR 1.25), ischemic heart disease (HR 1.24), cancer (HR 1.23), and osteoporosis (HR 1.20). In contrast, a medical history of dementia was associated with fewer acute hospitalisations (HR 0.78), see Table 4.

**Table 4:** Characteristics associated with acute hospital admission

		HR (95% CI) Unadjusted analysis	HR (95% CI) Fully adjusted analysis*
Sex			
	Female	1.00 (ref.)	1.00 (ref.)
	Male	**1.49 (1.32–1.69)**	**1.49 (1.32–1.69)**
Age			
	65–79 years	1.00 (ref)	1.00 (ref.)
	80–89 years	1.09 (0.95–1.26)	**1.17 (1.01–1.35)**
	90 + years	0.92 (0.78–1.10)	1.02 (0.86–1.22)
Selected morbidities			
	Cancer	**1.27 (1.09–1.47)**	**1.23 (1.06–1.43)**
	Diabetes	**1.39 (1.20–1.61)**	**1.32 (1.14–1.54)**
	Dementia	**0.79 (0.70–0.89)**	**0.78 (0.69–0.89)**
	Parkinson’s disease	1.07 (0.82–1.40)	1.04 (0.79–1.37)
	Alcohol abuse	1.05 (0.83–1.33)	0.95 (0.74–1.21)
	Schizophrenia, schizotypal and delusional disorders	1.25 (0.90–1.73)	1.21 (0.87–1.68)
	Mood disorders	1.02 (0.87–1.21)	1.04 (0.88–1.23)
	Anxiety	0.95 (0.73–1.25)	0.93 (0.70–1.23)
	Hypertension	**1.32 (1.17–1.50)**	**1.28 (1.13–1.45)**
	Ischemic heart disease	**1.36 (1.17–1.58)**	**1.24 (1.06–1.45)**
	Heart failure	**1.51 (1.26–1.82)**	**1.38 (1.14–1.67)**
	Atrial fibrillation	**1.37 (1.19–1.59)**	**1.25 (1.08–1.45)**
	Stroke	**1.19 (1.04–1.37)**	1.06 (0.92–1.22)
	COPD†/asthma	**1.32 (1.13–1.54)**	**1.30 (1.11–1.52)**
	Osteoporosis	1.11 (0.97–1.28)	1.20 **1.04–1.39)**

The cohort was divided into two groups; residents who experienced at least one acute hospital admission during the study period and residents with no acute admissions. The relation between specific characteristics of the residents and acute hospital admissions was analysed using Cox Regression, adjusting for the left-truncation set on 1st January 2018 and adjusting for competing risks by viewing it as censoring. Admission was the event of interest, and death due to any cause was the competing event preventing the resident from experiencing the event of interest.

* Adjusted for possible confounders concerning the given exposure based on clinical knowledge (see Additional file 3)

† Chronic obstructive pulmonary disease

Significant findings are in bold

## Discussion

To our knowledge, this is the first study since 2006 [29] to investigate factors associated with acute hospital admissions of CHRs, and it provides register-based documentation on the characteristics of the CHRs and their contacts to the secondary healthcare sector. Residents had a rate of 0.56 acute hospital admissions per resident-year. About three-quarters of the acutely admitted CHRs needed an in-hospital stay for at least 24 h. While one-third could be managed solely in the ED, more than half were referred to an in-hospital ward with a stay longer than 48 h. The primary diagnoses cover a range of acute diseases common in older adults admitted to hospital, such as pneumonia, urinary tract infection, and fracture of lower limbs. There was a high prevalence of readmissions (24.6%) and admission-related mortality (10.9% in-hospital and 13.0% 30 days post-discharge). Male sex was associated with acute hospital admissions, as was a medical history of various cardiovascular diseases, cancer, COPD/asthma, and osteoporosis. In contrast, a medical history of dementia was associated with fewer acute admissions.

### Rates of admissions

The comparison of rates of acute admissions with other studies is not straightforward. One reason is the conceptual differences in the definition of hospitalisations. In our study, we distinguished between emergency room visits and acute admissions. Emergency room visits were considered acute outpatient contacts when managed solely in the emergency room without needing further hospitalisation. All other unplanned hospital contacts were considered acute admissions. A Norwegian study with a similar definition reported an admission rate of 0.62 per person-year [6], comparable to this study’s finding of 0.56.

Other international studies often report on all ED contacts, including minor injuries managed in the emergency room [8, 10, 11, 30, 31]. This will naturally increase the rate of acute hospital referrals compared to our study. Two recent studies assessing all ED contacts reported an annual rate of referrals between 0.35 and 0.72 [30, 32]. However, a systematic review including older studies showed marked differences in annual rates of ED referrals per resident, varying from 0.20 to 1.50 [31]. These publications demonstrate the significant variation between studies complicating the comparison of results. The present study showed a rate of emergency room visits of 0.26, giving a total rate of acute hospital referrals of 0.82 per resident-year, which is within the range of international studies reporting on all ED contacts in total.

We found that almost one in four (24.6%) admissions resulted in acute readmission within 30 days from discharge. This is relatively high compared to international studies, reporting readmission rates of 6.1-7% within 30 days [30, 33], but may be explained by these studies counting all acute hospital referrals, including emergency room visits. As the present study excludes emergency room visits due to minor injuries, the remaining population is more critically ill than in other studies, leading to a higher prevalence of readmissions. A recent Danish study reported a rate of acute hospital reattendance of up to 23.8% within 30 days of an acute medical ED contact in geriatric patients with disability, polypharmacy, and comorbidity [34]. A possible explanation for the many readmissions of CHRs is a tendency to discharge CHRs earlier from Danish hospitals to reduce the risk of adverse events such as functional loss and hospital-acquired infections. Care homes are staffed with healthcare professionals who can monitor recently discharged residents and alert in case of deterioration. Another recent Danish study showed that CHRs had significantly shorter hospital stays compared to older citizens living at home but dependent on home care. Here, short-term admissions (< 48 h) comprised 20.4% of all admissions of CHRs and only 15.7% of admissions of dependent citizens [35]. Still, a readmission rate of 24.6% is high, and although the vulnerability and early discharge of CHRs may partly explain it, further studies are needed to investigate the reasons for readmissions.

Another reason complicating the comparison of studies on acute admissions from care homes is fundamental differences in the level of care in care home settings across and even within countries. Such a difference is seen in Britain, where care homes are divided into residential care homes that provide care and support 24 h a day and nursing homes that provide additional nursing care 24 h a day. A British study showed higher admission rates from residential homes compared to nursing homes [36]. Denmark’s care home referral practice means that only the most vulnerable citizens will achieve a long-term care home bed, regardless of financial means. This may influence the characteristics of the residents as well as the rates and reasons for acute admissions.

### Characteristics of care home residents

The high prevalence of selected morbidities emphasizes that CHRs have a substantial degree of complex diseases and multi-morbidity. Various methods for assessing morbidities are presented in the literature, but the results correlate to ours regarding most morbidities [5, 37]. Male residents had a higher disease burden and shorter survival after care home admission than females. We saw that the prevalence of most selected morbidities was generally higher amongst the younger age groups. A possible explanation for the decline in the prevalence of morbidities in old age is found in the definition of morbidities in the present study: Morbidities were based on all diagnoses from in- and outpatient hospital contacts for the past ten years (thus only covering hospital diagnoses) combined with the use of prescription medicines in the past year. To avoid an overestimation of the disease prevalence, ATC-codes were only included in the assessment of morbidities if the particular drug was solely indicated for the specific morbidity. Combined with the hospital-based ICD-10 codes, this will naturally lead to an underestimation of the prevalence of some selected morbidities, such as morbidities only diagnosed in the primary care sector (e.g., hypertension) and diseases never properly diagnosed (e.g., dementia, heart failure). The lower prevalence of morbidities among the oldest (age 90+) residents might suggest a tendency to refrain from initiating new outpatient examinations or termination of ongoing outpatient hospital contacts in the oldest, along with a tendency to withhold the oldest residents in the care home facility in case of acute illness, both leading to some underestimation of the disease prevalence. Another possible explanation is that the most multi-morbid residents die at earlier ages (selective mortality) leaving the relatively less multi-morbid older residents still alive. Other studies have shown similar patterns in the prevalence of morbidities with increasing age [3, 38].

### Time of admission

More than half of admissions were initiated during regular business hours, and three in four were initiated during weekdays. This result is in accordance with other studies [8, 10, 39]. As in many other countries, Danish care homes are better staffed during the daytime, and most care homes are only staffed with a nurse in the daytime on weekdays [7]. It is, therefore, more likely that the deteriorating health of a resident will be managed in this time span when the PCP is available. Still, many acute admissions occurred outside the PCP’s working hours, that is, in the late evening, overnight, or on weekends. A Canadian study showed an increased incidence of potentially preventable admissions during nights and on weekends [32]. Uncertainty with the trajectory in cases of acute illness and unfamiliarity with the resident’s medical history composes a challenge to on-call doctors. Improved information-sharing across healthcare sectors may help prevent some unnecessary or inappropriate hospital admissions.

In 2016 “care home physicians” were introduced in Denmark, where PCPs and local care homes were encouraged to join forces, which among other things, involved scheduled visits by the PCPs in the care homes. If the interprofessional collaboration between PCPs and care homes is strengthened, it may result in easier access to the PCP, greater familiarity between PCP, care home, and resident, and enhanced focus on medication. This could prevent the initiation of some acute hospital admissions during nights and weekends. Many Danish care homes now offer new residents an affiliation with the care home physician upon care home admission, and this arrangement is generally popular [40]. Further studies are needed to assess the effect of introducing care home physicians in Denmark [32].

### Duration of admission

A median length of stay of three days for all acute admissions and five days for those admitted to an in-hospital ward corresponds to other recent studies [6, 41]. We found that a third of all acute admissions were managed solely in the ED, with no need for referral to an in-hospital ward. This result also reflects reports from other studies [10, 11, 31]. Some short admissions managed solely in the ED might represent medical cases that could have been handled in the primary sector. However, acutely ill, multi-morbid older patients often present with unspecific complaints such as mental deterioration, delirium, general malaise, immobilisation, and falls, complicating correct diagnostics [42, 43]. Studies show that older patients presenting with unspecific complaints require increased use of diagnostic testing and procedures [44, 45], which are complex to manage in the primary sector. Increased access to and use of Point-of-Care Testing in the care home facilities could prevent some hospital referrals. The large proportion of admissions due to infections suggests that an intensified focus on tracking early signs of infection in the care homes could result in earlier initiation of relevant antibiotics and thereby increase the chances of successful treatment in the primary sector.

### Discharge diagnoses

The primary diagnoses at discharge from acute admissions corresponded to those found in other studies [11, 31], the most recurrent being pneumonia, urinary tract infections, and fall-related fractures. Hospital admissions of adults 65 years or older with these specific diagnoses are identified as potentially preventable both in Denmark and internationally [46–49]. Potentially preventable admissions are defined as hospitalisations due to conditions or events that could have been avoided altogether or managed in the primary sector without hospitalisation. However, the list of diagnoses considered to cause potentially preventable admissions do not account for the clinical complexity of acutely ill CHRs nor their many comorbidities, and measures of potentially preventable hospitalisations have not been validated in the care home population [47]. Even though these diagnoses are defined as potentially preventable, this is not always feasible in reality. For example, some admissions of CHRs with infections can be avoided if symptoms are recognised early, but this is more difficult or even impossible in the case of rapid onset and progressive development of a disease trajectory Also, a hospital admission is only preventable if a safe alternative is available. The Hospital-at-Home concept offers a way of avoiding some acute admissions from care homes. Here, hospital-level care is provided in the home by healthcare professionals as an alternative to acute admission [50], resulting in similar patient outcomes when compared to a traditional hospital admission [51, 52]. Studies show that improving and intensifying the care provided in the primary sector can effectively reduce hospital transfers and admissions from care homes [53, 54].

It is important to acknowledge that the decision to hospitalise is complex and influenced by factors other than the tentative diagnosis or clinical picture. Variations between different care home facilities and municipalities in the amount of nursing care, staffing, education, and previous experiences of staff all impact the decision to admit. Further studies are needed to investigate how organisational aspects within the municipalities and care home facilities influence the decision to admit CHRs in case of acute illness.

### Mortality

As in other studies, we found acute admissions to be associated with high mortality, with an in-hospital mortality of 10.9% and a mortality of 13.0% within 30 days post-discharge [10, 11]. The high mortality underlines how vulnerable and multi-morbid the CHRs are. Some of the deaths related to hospitalisation can also represent situations where residents are admitted to hospital at the very end of their lives and where end-of-life discussions with the next of kin and PCP have not been held. Improved interprofessional collaboration between PCPs and care homes would likely result in an enhanced continuity of care by the PCPs and an improved relationship between physicians, residents, and their next of kin, facilitating a more candid discussion on planning end-of-life care in the care home. This would likely decrease the number of inappropriate hospital transfers and in-hospital mortality.

### Factors associated with acute hospital admissions

This study found several factors associated with acute hospital admissions of CHRs. Residents who experienced at least one acute hospital admission during the study period had a higher prevalence of cardiovascular diseases (atrial fibrillation, hypertension, ischemic heart disease, and heart failure), cancer, COPD/asthma, and osteoporosis. However, the assessment of morbidities in the present study is only based on the use of prescribed medicine combined with hospital-based diagnoses. The results may be biased by the existence of CHRs who are only diagnosed with the selected morbidities in the PCPs’ records or even never properly diagnosed.

### Strengths and weaknesses

A significant strength of the present study is the relatively large and complete cohort of all CHRs in Southern Jutland, enriched with information on all hospital contacts through the highly valid Danish national health care registries. These study strengths enable us to provide baseline information on all residents, including those with no hospital admissions in the study period. However, studies based solely on register-based data are limited by the information not found in the registries, such as assessments of frailty or functional ability, information on morbidities not registered in hospital records but only by PCPs, or even never correctly diagnosed, or clinical decisions to “do-not-resuscitate” or “do-not-admit”.

Nevertheless, our findings contribute to the discussion on improving or preventing acute admissions from care home settings by providing an overview of the residents, their health characteristics, and contacts with the healthcare system. Though care home settings vary considerably across countries, the similarities of the results with other studies on the health characteristics of CHRs and their acute hospital admissions suggest that our findings could be helpful outside the Danish context. However, in-depth case studies on the pathway from care home to hospital are warranted to understand better which CHRs would benefit from acute hospital admission.

## Conclusion

In conclusion, this study is important to the ongoing discussion on improving or preventing care home residents’ acute admissions. The study highlights the characteristics of care home residents and their acute hospital admissions. Furthermore, our results indicate a direction for future studies targeting preventive efforts to reduce acute admissions from care home settings, such as strengthening the collaboration between primary care physicians and care homes, increased focus on planning end-of-life care, improved information-sharing across healthcare sectors, and increased access to and use of Point-of-Care Testing in care home facilities.

## Electronic supplementary material

Below is the link to the electronic supplementary material.

Supplementary Material 1: Appendix A

Supplementary Material 2: Supplementary table 1

Supplementary Material 3: Appendix B

Supplementary Material 4: Supplementary table 2

## Data Availability

The data that support the findings of this study are available from the Danish Health Data Authority but restrictions apply to the availability of these data, which were used under licens for the current study, and so are not publicly available. Data are however available from the authors upon a reasonable request and with permission of the Danish Health Data Authority.

## Abbreviations

ATC-codes: Anatomical Therapeutic Chemical Index
CHData: Care Home Data
CHRs: Care home residents
CI: Confidence interval
COPD: Chronic obstructive pulmonary disease
CPR number: Danish civil registration number
CRS: The Danish Civil Registration System
DNPR: The Danish National Patient Register
ED: Emergency department
ER: Emergency room
HR: Hazard ratio
ICD-10 codes: International Classification of Diseases
NPR: The Danish National Prescription Registry
PCP: Primary care physician
