# Supplementary material for: Associations between care home residents’ characteristics and acute hospital admissions – a retrospective, register-based cross-sectional study
Source: BMC Geriatr. 2023 Apr 18;23:234. doi: 10.1186/s12877-023-03895-1 (PMC10114422; doi:10.1186/s12877-023-03895-1)
Supplement: Supplementary file 1 — Supplementary Material 1: Appendix A [file 12877_2023_3895_MOESM1_ESM.docx]

Appendix A: ICD-10 codes and ATC-codes used to define comorbidities in Table 1.

| **Comorbidity** | **IDC-10 codes given within the past ten years from baseline** | **ATC-codes of prescription medicines within the past year from baseline** |
| --- | --- | --- |
| Cancer | C, D45-D47 | L01 |
| Diabetes | E10-E11, E13-E14 | A10 |
| Dementia | F00-F03, G30, G310B, G311, G318B, G318E, F1073, F1173, F1373, F1473, F1573, F1673, F1873, F1973 | N06D |
| Parkinson’s disease | F023, G20-G22 | N04BA, N04BB, N04BD, N04BX |
| Alcohol abuse | F101-F109 | N07BB |
| Schizophrenia, schizotypal and delusional disorders | F20-F29 | - |
| Mood disorders | F30-F34, F38-F39 | - |
| Anxiety | F40-F41, F43 | - |
| Hypertension | I10-I15 | - |
| Ischemic heart disease | I20-I25 | C01DA, C01DX16 |
| Heart failure | I099A, I110, I130, I132, I420, I426-I427, I429, I50 | - |
| Atrial fibrillation | I48 | C01AA |
| Stroke | I60-I64, I69 | - |
| COPD/asthma | J40-J47 | R03, a minimum of two dispensed prescriptions within one year |
| Osteoporosis | M80-M81 | G03XC01, H05AA02-H05AA03, M05BA-M05BB, M05BX04, M05BX06 |
