# Supplementary material for: Associations between care home residents’ characteristics and acute hospital admissions – a retrospective, register-based cross-sectional study
Source: BMC Geriatr. 2023 Apr 18;23:234. doi: 10.1186/s12877-023-03895-1 (PMC10114422; doi:10.1186/s12877-023-03895-1)
Supplement: Supplementary file 2 — Supplementary Material 2: Supplementary table 1 [file 12877_2023_3895_MOESM2_ESM.docx]

**Supplementary table 1: All primary discharge diagnoses from acute hospitalisations of care home residents in Southern Jutland in 2018-2019.**

| **Chapter** | **Title** | **Subcategories** | **ICD-10 codes** | **Total N (%)** | |
| --- | --- | --- | --- | --- | --- |
|  |  |  |  |  | **Hereof** |
| **I** | **Certain infectious and parasitic diseases** |  | **A00-B99** | **116 (6.9%)** | |
|  |  | Other bacterial diseases | A3-A4 |  | 100 (6.0%) |
|  |  | Other |  |  | 16 (1.0%) |
| **II** | **Neoplasms** |  | **C00-D48** | **10 (0.6%)** | |
| **III** | **Diseases of the blood and bloodforming organs and certain disorders involving the immune mechanism** |  | **D50-D89** | **18 (1.1%)** | |
| **IV** | **Endocrine, nutritional and metabolic diseases** |  | **E00-E90** | **99 (5.9%)** | |
|  |  | Diabetes mellitus | E10-E14 |  | 6 (0.4%) |
|  |  | Volume depletion | E86 |  | 62 (3.7%) |
|  |  | Other disorders of fluid, electrolyte and acid-base balance | E87 |  | 24 (1.4%) |
|  |  | Other |  |  | 7 (0.4%) |
| **V** | **Mental and behavioural disorders** |  | **F00-F99** | **93 (5.5%)** | |
|  |  | Dementia | F00-F03 |  | 44 (2.6%) |
|  |  | Delirium | F05 |  | 23 (1.4%) |
|  |  | Other |  |  | 26 (1.5%) |
| **VI** | **Diseases of the nervous system** |  | **G00-G99** | **25 (1.5%)** | |
|  |  | Parkinson’s’ disease | G20-G22 |  | 1 (0.1%) |
|  |  | Epilepsy and recurrent seizures | G40-G41 |  | 9 (0.5%) |
|  |  | Other |  |  | 15 (0.9%) |
| **VII** | **Diseases of the eye and adnexa** |  | **H00-H59** | **1 (0.1%)** | |
| **VIII** | **Diseases of the ear and mastoid process** |  | **H60-H95** | **0 (0.0%)** | |
| **IX** | **Diseases of the circulatory system** |  | **I00-I99** | **175 (10.4%)** | |
|  |  | Ischemic heart disease | I20-I25 |  | 17 (1.0%) |
|  |  | Pulmonary embolism | I26 |  | 14 (0.8%) |
|  |  | Conduction disorders | I44-I49 |  | 32 (1.9%) |
|  |  | Heart failure | I50 |  | 24 (1.4%) |
|  |  | Cerebrovascular diseases | I60-I69 |  | 43 (2.6%) |
|  |  | Other |  |  | 45 (2.7%) |
| **X** | **Diseases of the respiratory system** |  | **J00-J99** | **371 (22.1%)** | |
|  |  | Pneumonia | J13-J18 |  | 221 (13.2%) |
|  |  | Chronic lower respiratory diseases | J40-J47 |  | 51 (3.0%) |
|  |  | Pneumonitis due to solids and liquids | J69 |  | 29 (1.7%) |
|  |  | Respiratory failure, not elsewhere classified | J96 |  | 54 (3.2%) |
|  |  | Other |  |  | 16 (1.0%) |
| **XI** | **Diseases of the digestive system** |  | **K00-K93** | **112 (6.7%)** | |
|  |  | Diseases of oesophagus, stomach and duodenum | K20-K31 |  | 23 (1.4%) |
|  |  | Other diseases of intestines | K55-K64 |  | 38 (2.3%) |
|  |  | Disorders of gallbladder, biliary tract and pancreas | K80-K87 |  | 24 (1.4%) |
|  |  | Other |  |  | 27 (1.6%) |
| **XII** | **Diseases of the skin and subcutaneous tissue** |  | **L00-L99** | **9 (0.5%)** | |
| **XIII** | **Diseases of the musculoskeletal system and connective tissue** |  | **M00-M99** | **10 (0.6%)** | |
| **XIV** | **Diseases of the genitourinary system** |  | **N00-N99** | **146 (8.7%)** | |
|  |  | Renal failure | N17-N19 |  | 10 (0.6%) |
|  |  | Urinary tract infection | N30, N390 |  | 111 (6.6%) |
|  |  | Other |  |  | 25 (1.5%) |
| **XV** | **Pregnancy, childbirth and the puerperium** |  | **O00-O99** | **0 (0.0%)** | |
| **XVI** | **Certain conditions originating in the perinatal period** |  | **P00-P96** | **0 (0.0%)** | |
| **XVII** | **Congenital malformations, deformations and chromosomal abnormalities** |  | **Q00-Q99** | **1 (0.1%)** | |
| **XVIII** | **Symptoms, signs and abnormal clinical and laboratory findings, not elsewhere classified** |  | **R00-R99** | **162 (9.7%)** | |
|  |  | Symptoms involving circulatory and respiratory systems | R00-R09 |  | 34 (2.0%) |
|  |  | Symptoms involving digestive system and abdomen | R10-R19 |  | 34 (2.0%) |
|  |  | Symptoms involving nervous and musculoskeletal systems | R25-R29 |  | 17 (1.0%) |
|  |  | Symptoms involving cognition, perception, emotional state and behaviour | R40-R46 |  | 12 (0.7%) |
|  |  | General symptoms and signs | R50-R69 |  | 51 (3.0%) |
|  |  | Other |  |  | 14 (0.8%) |
| **XIX** | **Injury, poisoning and certain other consequences of external causes** |  | **S00-T98** | **218 (13.0%)** | |
|  |  | Fractures in lower limbs | S720-S729  S820-S829  S92 |  | 119 (7.1%) |
|  |  | Other fractures | S02, S098B, S12, S22, S32, S42, S52, S62 |  | 26 (1.5%) |
|  |  | Intracranial injury | S06 |  | 16 (1.0%) |
|  |  | Other |  |  | 57 (3.4%) |
| **XX** | **External causes of morbidity and mortality** |  | **V01-Y98** | **0 (0.0%)** | |
| **XXI** | **Factors influencing health status and contact with health services** |  | **Z00-Z99** | **112 (6.7%)** | |
|  |  | Medical observation and evaluation for suspected diseases and conditions, ruled out. | Z03 |  | 81 (4.8%) |
|  |  | Other |  |  | 31 (1.8%) |
| **XXII** | **Codes for special purposes** |  | **U00-U85** | **0 (0.0%)** | |
|  | Total |  |  | **1678** | |
