# Supplementary material for: Associations between care home residents’ characteristics and acute hospital admissions – a retrospective, register-based cross-sectional study
Source: BMC Geriatr. 2023 Apr 18;23:234. doi: 10.1186/s12877-023-03895-1 (PMC10114422; doi:10.1186/s12877-023-03895-1)
Supplement: Supplementary file 3 — Supplementary Material 3: Appendix B [file 12877_2023_3895_MOESM3_ESM.docx]

Appendix B: List of possible confounders adjusted in relation to the given exposure in Table 4.

| **Exposure** | **Possible confounders** |
| --- | --- |
| Sex | - |
| Age-group | Sex |
| Cancer | Age group, sex, alcohol abuse, diabetes, COPD/asthma |
| Diabetes | Age group, sex, schizophrenia, schizotypal and delusional disorders, alcohol abuse |
| Dementia | Stroke, alcohol abuse, age group, Parkinson’s disease |
| Parkinson's disease | Sex, age group |
| Alcohol abuse | Sex, age group, schizophrenia, schizotypal and delusional disorders, mood disorders, anxiety |
| Severe psychiatric diseases | Dementia, Parkinson’s disease, mood disorders, anxiety, alcohol abuse |
| Mild psychiatric diseases | Dementia, Parkinson’s disease, schizophrenia, schizotypal and delusional disorders, anxiety, alcohol abuse |
| Anxiety | Dementia, Parkinson’s disease, schizophrenia, schizotypal and delusional disorders, mood disorders, alcohol abuse |
| Hypertension | Sex, age group, diabetes |
| Ischemic heart disease | Sex, age group, diabetes, alcohol abuse, COPD/asthma |
| Heart failure | Ischemic heart disease, age group, diabetes, hypertension, alcohol abuse |
| Atrial fibrillation | Age group, hypertension, alcohol abuse, sex, ischemic heart disease |
| Stroke | Age group, sex, atrial fibrillation, hypertension |
| COPD/asthma | Sex, alcohol abuse, schizophrenia, schizotypal and delusional disorders, mood disorders, anxiety |
| Osteoporosis | Sex, age group, COPD/asthma |
